# Mu Opioid Splice Variant MOR-1K Contributes to the Development of Opioid-Induced Hyperalgesia

**DOI:** 10.1371/journal.pone.0135711

**Published:** 2015-08-13

**Authors:** Folabomi A. Oladosu, Matthew S. Conrad, Sandra C. O’Buckley, Naim U. Rashid, Gary D. Slade, Andrea G. Nackley

**Affiliations:** 1 Center of Pain Research and Innovation, University of North Carolina–Chapel Hill, Chapel Hill, NC, United States of America; 2 Neuroscience Program, University of Illinois at Urbana-Champaign, Urbana, IL, United States of America; 3 Department of Biostatistics, University of North Carolina–Chapel Hill, Chapel Hill, NC United States of America; University of Kentucky Medical Center, UNITED STATES

## Abstract

**Background:**

A subset of the population receiving opioids for the treatment of acute and chronic clinical pain develops a paradoxical increase in pain sensitivity known as opioid-induced hyperalgesia. Given that opioid analgesics are one of few treatments available against clinical pain, it is critical to determine the key molecular mechanisms that drive opioid-induced hyperalgesia in order to reduce its prevalence. Recent evidence implicates a splice variant of the mu opioid receptor known as MOR-1K in the emergence of opioid-induced hyperalgesia. Results from human genetic association and cell signaling studies demonstrate that MOR-1K contributes to decreased opioid analgesic responses and produces increased cellular activity *via* G_s_ signaling. Here, we conducted the first study to directly test the role of MOR-1K in opioid-induced hyperalgesia.

**Methods and Results:**

In order to examine the role of MOR-1K in opioid-induced hyperalgesia, we first assessed pain responses to mechanical and thermal stimuli prior to, during, and following chronic morphine administration. Results show that genetically diverse mouse strains (C57BL/6J, 129S6, and CXB7/ByJ) exhibited different morphine response profiles with corresponding changes in *MOR-1K* gene expression patterns. The 129S6 mice exhibited an analgesic response correlating to a measured decrease in *MOR-1K* gene expression levels, while CXB7/ByJ mice exhibited a hyperalgesic response correlating to a measured increase in *MOR-1K* gene expression levels. Furthermore, knockdown of *MOR-1K* in CXB7/ByJ mice *via* chronic intrathecal siRNA administration not only prevented the development of opioid-induced hyperalgesia, but also unmasked morphine analgesia.

**Conclusions:**

These findings suggest that MOR-1K is likely a necessary contributor to the development of opioid-induced hyperalgesia. With further research, MOR-1K could be exploited as a target for antagonists that reduce or prevent opioid-induced hyperalgesia.

## Introduction

Opioids are the most commonly used treatment for moderate to severe clinical pain, with 259 million prescriptions written within the United States in 2012 [1]. Paradoxically, opioids can also produce pain. This abnormal phenomenon, known as opioid-induced hyperalgesia (OIH), is defined by increased pain sensitivity that occurs following acute or chronic opioid administration and is distinct from the originally reported pain [2]. OIH is especially prevalent in individuals who suffer from chronic pain, affecting up to 30% of individuals that use opioids to treat their pain [2,3]. OIH is often confused with tolerance to and withdrawal from the administered opioid treatment. Unlike opioid tolerance, which is a decreased analgesic efficacy of the same opioid dose over time, OIH can manifest at any opioid dose during acute or chronic administration [4]. Unlike opioid withdrawal, which is characterized by a hyperalgesic response following abrupt opioid cessation, OIH manifests during as well as following opioid administration [4]. Mistaking OIH for opioid tolerance or withdrawal results in inadequate treatment options that may intensify the reported pain. Thus, it is critical to identify the molecular mechanisms underlying OIH.

Accumulating evidence indicates that MOR-1K, a functional splice variant of the canonical mu opioid receptor (MOR-1), may contribute to the emergence of OIH. MOR-1K is a truncated six transmembrane g-protein coupled receptor (GPCR) lacking a N-terminus transmembrane due to the absence of exon 1 within its mRNA transcript [5]. Replacing exon 1 are exon 11, which provides an alternative translation start site in several MOR-1 splice variants, and exon 13, which is unique to the *MOR-1K* transcript. The MOR-1 transcript, which encompasses exons that encode for MOR-1K, is highly conserved across species, with a 91% nucleotide sequence homology between human and mouse. Results from a human genetic association study demonstrated that a single nucleotide polymorphism within exon 13 of the human *MOR-1K* transcript is associated with increased pain sensitivity and blunted morphine efficacy [6]. Subsequent *in vitro* studies demonstrated that MOR-1K exhibits signaling properties distinct from its parent receptor MOR-1. MOR-1 utilizes G_i/o_ protein to inhibit cyclic adenosine monophosphate (cAMP) levels and intracellular calcium levels to produce cellular inhibition of pronociceptive cells. In contrast, MOR-1K couples to G_s_ protein, leading to increased cAMP production and intracellular calcium levels, thus promoting cellular excitation [7]. Previous studies have shown that G_s_-dependent increases in intracellular calcium *via* cAMP production and protein kinase A activation play a critical role in central sensitization [8] and the development of inflammatory, neuropathic, and functional pain [9]. The utilization of G_s_ signaling by MOR-1K suggests that the receptor may also contribute to central sensitization associated with OIH.

Given the receptor’s genetic association with increased pain sensitivity and its excitatory signaling profile, we hypothesize MOR-1K may contribute to OIH in genetically susceptible individuals. Here, we evaluate MOR-1K in the development of OIH using three genetically diverse mouse strains alongside small interfering RNA (siRNA) knockdown of *MOR-1K*. Our results demonstrate that OIH is associated with increased *MOR-1K* gene expression levels in a strain-specific manner. Disrupting the increase in *MOR-1K* gene expression levels *via* chronic intrathecal (i.t.) siRNA administration not only hinders the development of OIH, but also increases morphine analgesic efficacy. Collectively, these findings demonstrate that MOR-1K is likely a key contributor to OIH.

## Materials & Methods

### Ethical Statement

All procedures within this study were approved by the University of North Carolina Animal Care and Use Committee (permit number: 12–319) and adhered to the guidelines of the Committee for Research and Ethical Issues of the International Association of the Study of Pain (http://www.iasp-pain.org/Education/Content.aspx?ItemNumber=1217). All surgeries were performed under isofluorane anesthesia, and all efforts were made to minimize suffering.

### Animals

Male and female C57BL/6J (http://jaxmice.jax.org/strain/000664.html) and CXB7/ByJ (http://jaxmice.jax.org/strain/000357.html) mice were obtained from Jackson Labs (Bar Harbor, ME) while 129S6 (http://www.taconic.com/129SVE) mice were obtained from Taconic (Germantown, NY). All mice were 8–12 weeks old, weighed 20–30 g, were maintained under 12-hour light/dark cycle, and were fed *ad libitum*.

### Drugs and Chemicals

Morphine sulfate (Sigma, MO) was dissolved in 0.9% sterile saline (Hospira, IL). Doses of 10 mg/kg, 20 mg/kg, or 40 mg/kg were administered *via* subcutaneous (s.c.) injection in a volume determined by animal weight (1μl/g). Fluorescein-tagged exon 13-antisense siRNA [5’-UCA GUC UUU AUC AGC UCA CCG CCA-3’] or fluorescein-tagged exon 13-sense siRNA (Midland Certified Reagent Co., OH) [5’-AGU CAG AAA UAG UCG AGU GGC GGU-3’] in artificial cerebrospinal fluid was administered at a rate of 0.5μl/hr; 0.291μg/hr/day for a duration of 7 days *via* Alzet osmotic mini-pump (Durect, CA) connected to an i.t. catheter (Durect, CA). Previous studies have successfully administered siRNA in this fashion as well [10–12]. Sense siRNA was chosen as a negative control as it is related to the target mRNA sequence of interest but does not affect target mRNA expression [13].

### Experimental Design

#### Experiment 1: The effects of chronic morphine administration on pain behavior and gene expression

Prior to chronic morphine administration, C57BL/6J, CXB7/ByJ, and 129S6 mice (N = 96; 8 males and 8 females per experimental condition) were assessed for baseline responses to mechanical and thermal heat stimuli. Following baseline assessments, mice received morphine similar to the murine OIH protocol described by Liang et al [14]. Briefly, mice received vehicle (sterile saline) or escalating doses of morphine (10 mg/kg, 20 mg/kg, 20 mg/kg, and 40 mg/kg) twice daily (8am and 6pm) *via* s.c. injection on days 1, 2, 3, and 4, respectively. Behavioral responses were evaluated prior to and immediately following the 8am injection on days 1–4 and at 8am on days 5–7. Gene expression levels were measured in tissues collected from separate groups of C57BL/6J, 129S6, and CXB7/ByJ mice (N = 84; 3–4 males and 3–4 females per experimental condition) sacrificed on day 0, on days 1 or 4 following the 8am injection, or on day 5 at 8am.

#### Experiment 2: The effects of MOR-1K exon 13 siRNA knockdown on OIH and MOR-1K gene expression

Prior to chronic morphine administration, CXB7/ByJ mice (N = 48; 8 males and 8 females per experimental condition) were assessed for baseline responses to mechanical stimuli. Following baseline assessments, mice underwent surgery for chronic i.t. administration of antisense exon 13 siRNA or sense exon 13 siRNA. Mice were anesthetized with 5% isofluorane and maintained at 2–3% isofluorane during i.t. catheter implantation, modified from Yaksh and Rudy protocol [15]. A separate group of mice also underwent surgery for a sham procedure. The sham procedure, involving skin incision and muscle dissection without breakage of the arachnoid membrane to cause leakage of cerebral spinal fluid, was deemed appropriate to control for any postoperative pain. One day following surgery, mice from antisense, sense, and sham conditions began to receive either vehicle (sterile saline) or escalating doses of morphine as described above. *MOR-1K* gene expression levels were measured in tissues collected from separate groups of CXB7/ByJ mice (N = 84; 3–4 males and 3–4 females per experimental condition) sacrificed on day 0, on days 1 or 4 following the 8am injection, or on day 5 at 8am.

### Behavior

#### Assessment of Paw Withdrawal Threshold, Mechanical Allodynia, and Mechanical Hyperalgesia

Mice were handled and habituated to the testing environment for 4 days prior to baseline assessments. On test days, mice were placed in plexiglass cages positioned over an elevated wire mesh platform and habituated to the environment for 20 minutes. Paw withdrawal threshold in response to a series of 9 von Frey filaments (with bending forces of 0.03, 0.07, 0.17, 0.40, 0.70, 1.19, 1.50, 2.05, 3.63g; Stoeling, IL) was assessed using the “up-down” method [16], starting with a filament with bending force of 0.70 g. In the absence of a paw withdrawal response, an incrementally stronger filament was presented and in the event of a paw withdrawal, an incrementally weaker filament was presented. After the initial response threshold was crossed, this procedure was repeated in order to obtain a total of six responses in the immediate vicinity of the threshold. The pattern of withdrawals and absence of withdrawals were noted together with the terminal filament used in the series of six responses. The 50% of the paw withdrawal threshold is calculated as (10^[X^_f_^+kδ]^)/10,000, where *X*_f_ = value (in log units) of the final von Frey hair used; *k* = tabular value of pattern of positive (*X*) and negative (*O*) responses, and *δ* = mean difference (in log units) between stimuli. Mechanical allodynia was assessed by presenting a filament with bending force of 0.40 g to the hind paw 10 times for a duration of 1 s with an inter-stimulus interval of 1 s. A significant increase in the percentage frequency of paw withdrawal ([# of paw withdrawals/10] x 100) was defined as mechanical allodynia. Mechanical hyperalgesia was assessed in the same manner, using a filament with a bending force of 1.50 g.

#### Assessment of Thermal Heat Hyperalgesia

Thermal heat hyperalgesia was evaluated using the hot plate method [17]. Mice were placed on a hot plate (Columbus Instruments, OH) maintained at a temperature of 51.5°C for one minute. Each session was videotaped and the total number of aversive responses (paw licks, paw flicks, and jumps) was measured.

### Assessment of Gene Expression Levels

Discrete brain and spinal cord samples were collected on days 0, on day 1 and 4 following the 8am morphine administration, and on day 5, 24 hours following morphine cessation. Total RNA from discrete brain regions (medulla, pons, periaqueductal grey, thalamus, hypothalamus, striatum, nucleus accumbens, and frontal lobe), and spinal cord was purified using 1mL TRIzol (Life Technologies, NY) for each tissue sample. Samples were immediately homogenized using a Pro200 homogenizer (Pro Scientific, CT) or Precellys 24 Homogenizer (Bertin Technologies, France) and all subsequent RNA purification steps were performed according to the TRIzol manufacturer recommendations. Purified RNA samples were treated with TURBO DNA-free (Life Technologies, NY) per manufacturer protocol and concentrations were determined using a Nanodrop-1000 (Thermo Scientific, DE) and reverse transcribed using Transcriptor First Strand cDNA Synthesis kit (Roche, Switzerland) where necessary. Fast Start Universal SYBR Green Master with Rox (Roche, Switzerland) or Power SYBR Green RNA-to-CT 1-Step (Life Technologies, NY) were respectively used per manufacturer protocols to amplify cDNA and RNA, per manufacturer protocols. A 7900HT Fast Real-Time PCR system (Life Technologies, NY) and a StepOnePlus Real-Time PCR system (Life Technologies, NY) were respectively used for measuring cDNA or RNA transcripts amplification. The following primers were used for the detection of the following exon-11 containing MOR-1 splice variants: MOR-1K forward (TCCCCTCTTGAGTGTGACTAATGTC) and reverse (GCCAGAGCAAGGTTGAAAATG); MOR-1L forward (CAGAGCAAGGTTGAAAATGTAGATG) and reverse (AAATCAAAATAGAAAATGGGCTAAGG); MOR-1T forward (GAGCCACATGGAATTGCCTCTGTA) and reverse (GCATCTGCCAGAGCAAGGTTGAAA); forward (GGGCCGATGATGGAAGCTTTCTCTAA) and reverse (GCATCTGCCAGAGCAAGGTTGAAA) primers for splice variants that contain exons 11 and 2. Expression of the target genes was normalized to housekeeping genes RPL7 [forward (TCAATGGAGTAAGCCCAAAG) and reverse (CAAGAGACCGAGCAATCAA)] or GAPDH [forward (TGAAGGTCGGAGTCAACGGATTTGGT) and reverse (CATGTGGGCCATGAGGTCCACCAC)] using the 2^(-ΔΔCT)^ method. All primers were purchased from Integrated DNA Technologies (CA).

### Statistical Analysis

Baseline responses for paw withdrawal threshold, mechanical allodynia, and mechanical hyperalgesia for each strain were analyzed using one-way ANOVA followed by Bonferroni correction. Subsequent behavioral responses following morphine administration were then analyzed using two-way repeated measures ANOVA with Bonferroni correction for multiple comparisons. Gene expression was analyzed using a linear mixed model, where the log (10) gene expression of each tissue-replicate assay was the dependent variable and strain was the random effect. For all tests, the criterion for statistical significance level was *p<*0.05. Statistical analyses were performed using Prism (GraphPad Software, CA)

## Results

### Strains demonstrate divergent baseline pain profiles

First, we sought to establish baseline pain responses to mechanical stimuli in C57BL/6J, 129S6, and CXB7/ByJ mice. The C57BL/6J strain was chosen as it is the most studied classic inbred strain, the 129S6 strain was chosen because of its reported resistance to opioid tolerance [18], and the CXB7/ByJ strain was chosen because of its reported decrease in *MOR-1* gene expression [19]. For all three strains, male and female mice demonstrated similar behavioral differences to mechanical and thermal heat stimuli (S1 Fig); hence all behavioral data was pooled together for analysis.

**Fig 1:**
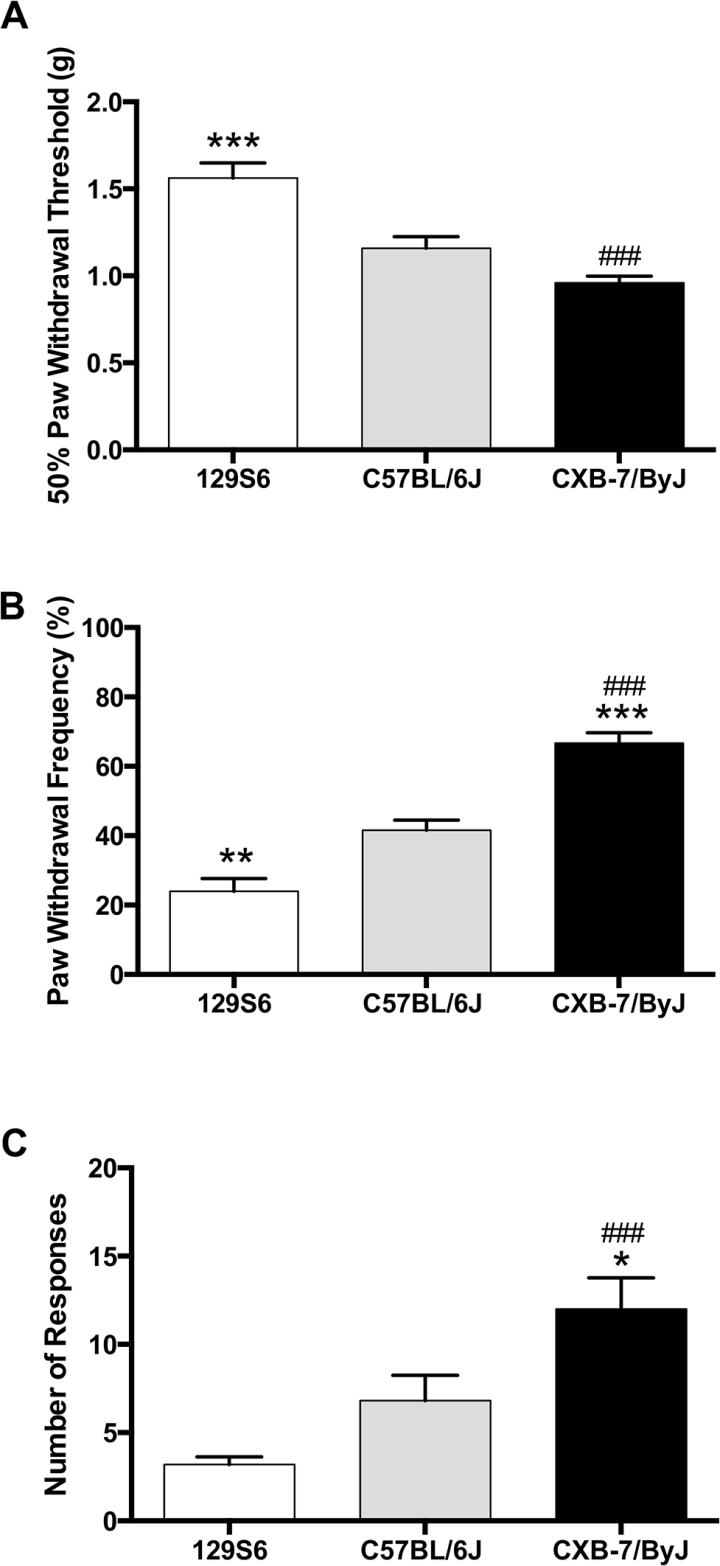
Strains exhibit divergent behavioral responses to mechanical stimuli at baseline. Compared to C57BL/6J mice, 129S6, and CXB7/ByJ mice exhibit differences in (A) paw withdrawal threshold, (B) the number of responses to repeated presentation of a noxious mechanical stimulus sensitivity, and (C) the number of responses to continuous thermal heat. *N* = 15-16/group. Data expressed as mean ± SEM. ***p<0.001, **p<0.01, *p<0.05 different from C57BL/6J. ^###^ *p*<0.001 different from 129S6.

Our results showed that the strains exhibited baseline differences in mechanical (Fig 1A, F_(2,43)_ = 20.56, *p*<0.0001; Fig 1B, F_(2,43)_ = 43.77, *p*<0.0001), and thermal heat pain sensitivity (Fig 1C; F_(2,43_ = 10.64, *p* = 0.0002). Compared to the classic C57BL/6J inbred strain, 129S6 mice were less pain sensitive, exhibiting higher paw withdrawal thresholds and fewer responses to a noxious mechanical stimulus or to thermal heat. In contrast, CXB7/ByJ mice were more pain sensitive, exhibiting lower paw withdrawal thresholds and increased responses to innocuous mechanical or thermal heat stimuli.

### Strains demonstrate divergent pain profiles in a chronic morphine administration paradigm

**Fig 2:**
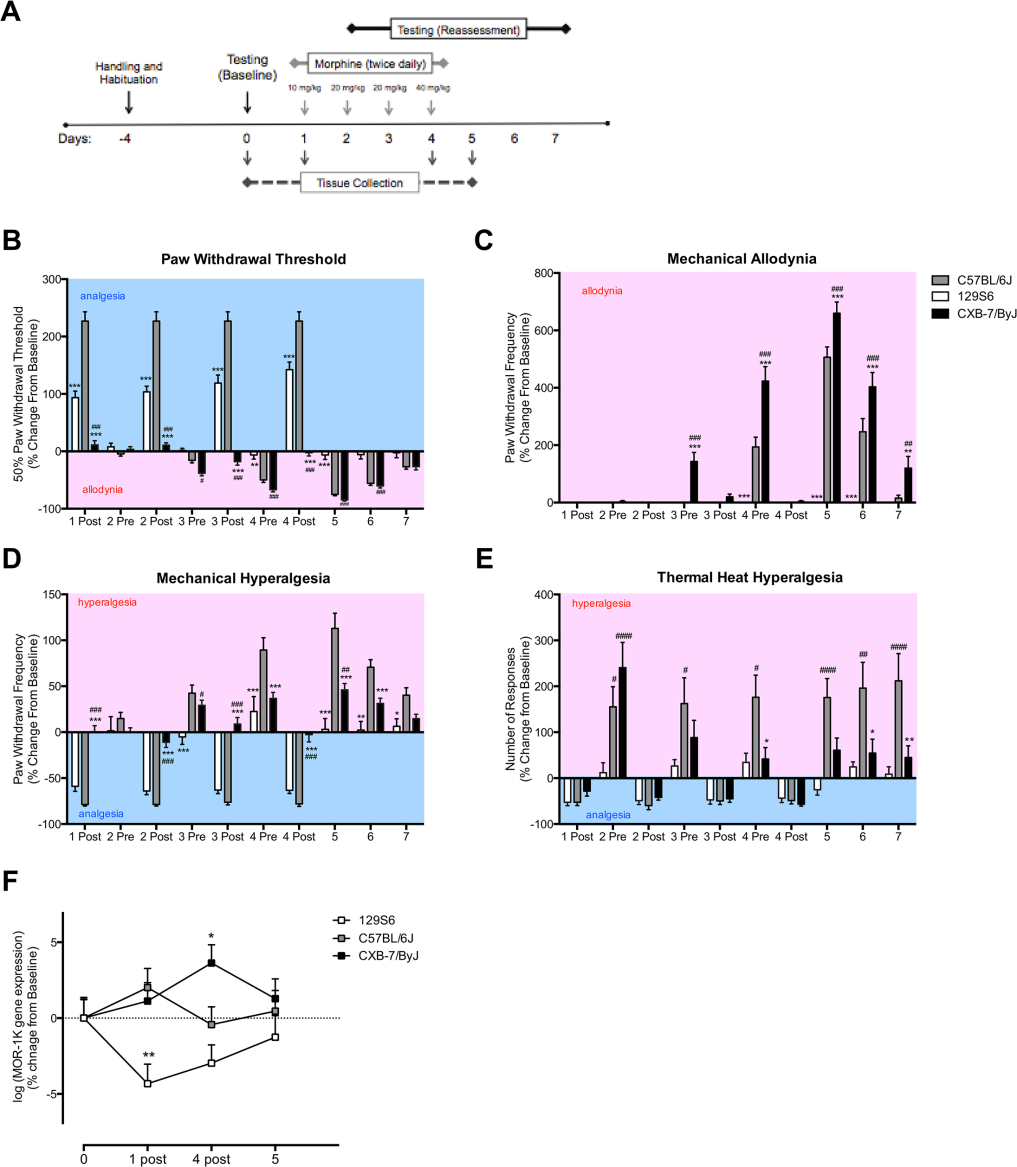
Strains exhibit divergent morphine-dependent analgesic and allodynic/hyperalgesic profiles with corresponding changes in *MOR-1K* gene expression. (A) A timeline of chronic morphine administration and the assessments of pain behavior and gene expression are shown. (B-E) Compared to C57BL/6J, 129S6 mice exhibit increased paw withdrawal thresholds and fail to develop allodynia or hyperalgesia. In contrast, CXB7/ByJ exhibit decreased paw withdrawal thresholds and increased allodynia and hyperalgesia. (F) 129S6 mice exhibit decreased *MOR-1K* gene expression levels on days 1 and 4, corresponding to their analgesic behavioral profile. In contrast, CXB7/ByJ mice exhibit increased *MOR-1K* gene expression levels on day 4, corresponding to their hyperalgesic behavioral profile. For behavioral graphs, blue background indicates a decrease in pain sensitivity (analgesia), and red background indicates an increase in pain sensitivity (allodynia/hyperalgesia). Panels B-E: *N* = 15-16/group. Data expressed as mean ± SEM. ***p<0.001, **p<0.01, *p<0.05 different from C57BL/6J. ^###^ *p<*0.001, ^##^ *p*<0.01, ^#^ *p*<0.05 different from 129S6. Panel F: *N* = 7/group. Data expressed as means ± SEM. *p<0.05 different from baseline.

Next, we sought to examine the strains’ pain responses following morphine administration using a modified murine paradigm for OIH [14]. We assessed behavioral responses to mechanical and thermal heat stimuli on days 1–4 during chronic morphine administration and after morphine cessation on days 5–7 (Fig 2, S2 Fig). When examining percent change from baseline responses, C57BL/6J, 129S6, and CXB7/ByJ mice exhibited differences in paw withdrawal thresholds over time (Fig 2B; F_(18,430)_ = 45.38, *p*<0.0001). C57BL/6J mice showed robust analgesia following morphine administration on days 1–4, then developed allodynia on day 4 prior to morphine administration and on days 5 and 6 following morphine cessation. When compared to C57BL/6J mice, 129S6 mice also showed analgesia following morphine administration on days 1–4, albeit to a lesser degree than C57BL/6J mice, however failed to develop allodynia at later time points (*p*<0.001). In contrast, CXB7/ByJ mice failed to show analgesia following morphine administration on days 1–4, but did develop allodynia prior to morphine administration on days 3 and 4 and following morphine cessation on days 5 and 6 (*p*<0.001). Of note, CXB7/ByJ mice also exhibited allodynia following morphine administration on day 3 (*p*<0.001).

Strains also exhibited differences in their response to repeated presentation of a normally innocuous punctate mechanical stimulus over time (Fig 2C; F_(18,430)_ = 32.88, *p*<0.0001). C57BL/6J mice developed mechanical allodynia prior to morphine administration on day 4 and following morphine cessation on days 5 and 6. Consistent with the paw withdrawal threshold data, 129S6 mice failed to develop mechanical allodynia. CXB7/ByJ mice, however, demonstrated the highest degree of mechanical allodynia, evident prior to morphine administration on days 3 and 4 and following morphine cessation on days 5–7 (*p*<0.001).

Similarly, strains exhibited differences in their response to repeated presentation of a normally noxious punctate mechanical stimulus over time (Fig 2D; F_(18,430)_ = 15.19, *p*<0.0001). C57BL/6J mice showed analgesia following morphine administration on days 1–4, then developed mechanical hyperalgesia prior to morphine administration on days 3 and 4 as well as following morphine cessation on days 5–7. 129S6 mice exhibited analgesia following morphine administration on days 1–4 (*p*<0.001), however failed to develop mechanical hyperalgesia at later time points. In contrast to the other strains, CXB7/ByJ mice failed to show analgesia following morphine administration, but did develop mechanical hyperalgesia prior to morphine administration on days 3 and 4 as well as following morphine cessation on days 5 and 6 (*p*<0.001). Consistent with their changes in mechanical allodynia, CXB7/ByJ mice also exhibited mechanical hyperalgesia following morphine administration on day 3 (*p*<0.001). When assessed for behavioral responses to mechanical stimuli, saline treated controls did not exhibit any changes in paw withdrawal threshold or demonstrate mechanical allodynia or hyperalgesia (S3 Fig).

Finally, strains differed over time with respect to their response to thermal heat (Fig 2E; F_(18,430)_ = 3.906, *p*<0.0001). When compared to 129S6 mice, C57BL/6J mice developed thermal heat hyperalgesia starting on day 2 prior morphine administration and following morphine cessation (*p*<0.001). CXB7/ByJ mice also developed develop thermal heat hyperalgesia on day 2 and 4 prior to morphine administration and following morphine cessation.

### *MOR-1K* gene expression levels parallel OIH profiles

To determine the relationship between OIH pain profiles and *MOR-1K* gene expression, spinal cord and discrete brain tissues were collected from separate groups of mice at times points corresponding to maximal analgesia and hyperalgesia. Changes in *MOR-1K* gene expression levels across the discrete brain and spinal cord tissues were not significantly different, and thus were normalized and pooled together (S4–S6 Figs). C57BL/6J, 129S6, and CXB7/ByJ mice exhibited divergent *MOR-1K* gene expression levels that paralleled their behavioral profiles (Fig 2F; *p* = 0.011). Compared to C57BL/6J mice, 129S6 mice demonstrated decreased *MOR-1K* gene expression levels on days 1 and 4 (*p*<0.05), in line with their analgesic responses. In contrast, CXB7/ByJ mice demonstrated increased *MOR-1K* gene expression levels on day 4 (*p*<0.05), in line with their hyperalgesic responses. Interestingly, *MOR-1K* gene expression levels for both 129S6 and CXB7/ByJ mice returned to near-baseline levels following the cessation of morphine treatment on day 5, suggesting that the observed differences were indeed morphine-dependent. To determine the potential involvement of other MOR-1 splice variants, we measured gene expression levels of other exon-11 containing splice variants (MOR-1G, MOR-1H, MOR-1I, MOR-1J, MOR-1L, MOR-1M, MOR-1N and MOR-1T). The splice variants did not exhibit significant changes in gene expression levels due to morphine administration (S7 Fig).

### Sustained delivery of *MOR-1K* exon 13 antisense siRNA prevents OIH

**Fig 3:**
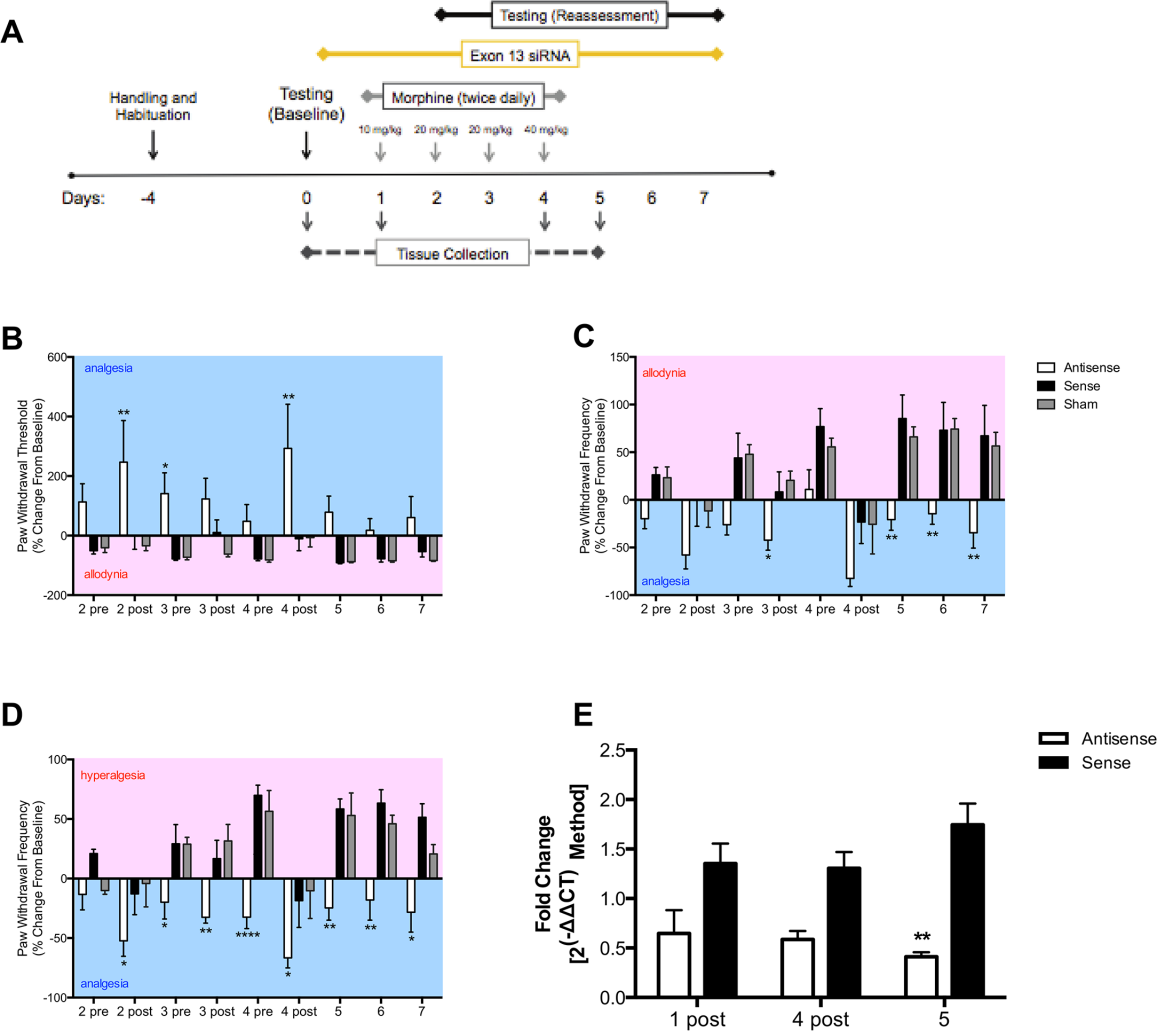
CXB7/ByJ mice treated with exon 13 antisense siRNA fail to develop OIH. (A) A timeline of sustained i.t. siRNA delivery, chronic morphine administration, and assessments of pain behavior and gene expression is shown. CXB7/ByJ mice receiving antisense siRNA exhibit analgesia following morphine administration and cessation, and fail to exhibit (B) decreased paw withdrawal thresholds, or increased responses to (C) repeated presentation of an innocuous or (D) noxious mechanical stimulus. In contrast, mice receiving sense siRNA or sham exhibited analgesia following morphine administration, which was then followed by allodynia/hyperalgesia on days 5–7. (E) CXB7/ByJ mice receiving antisense siRNA exhibit decreased *MOR-1K* gene expression levels in the spinal cord as compared to those receiving sense siRNA. For all behavioral graphs, blue background indicates a decrease in pain sensitivity (analgesia), and red background indicates an increase in pain sensitivity (allodynia/hyperalgesia). Panels B-D: *N* = 6-9/group. Data expressed as mean ± SEM.****p<0.0001, ***p<0.001, **p<0.01, *p<0.05 different from Sham. Panel E: *N =* 3-4/group. **p<0.01 different from Sense.

The observed correlation between strain-specific pain profiles and *MOR-1K* gene expression levels suggests that MOR-1K contributes to OIH in genetically susceptible mice. To determine whether MOR-1K is required for the development of OIH in CXB7/ByJ mice, we employed siRNA knockdown. Male and female CXB7/ByJ mice demonstrated similar behavioral phenotypes (S8 Fig); thus, all behavioral data was pooled together for analysis. Within the OIH murine paradigm, we found that sustained i.t. delivery of exon 13 antisense siRNA prevented the development of mechanical allodynia (Fig 3B; F_(2,189)_ = 24.69, *p*<0.0001, Fig 3C; F_(2,189)_ = 37.63, *p*<0.0001) and mechanical hyperalgesia (Fig 3D; F_(2,189)_ = 54.92, *p<*0.0001). In contrast, sustained administration of exon 13 sense siRNA or sham surgery did not prevent the development of mechanical pain sensitivity. Of note, we also found that sustained i.t. delivery of the antisense siRNA unmasked morphine analgesia in CXB7/ByJ mice not observed in sense and sham mice or saline-treated controls (S9 and S10 Figs) A possible concern with sustained i.t. delivery is the development of inflammation and gliosis in the spinal cord in proximity with the catheter tip [20,21], and its effects on behavioral assessment. Given that CXB7/ByJ mice receiving sense siRNA and sham surgery displayed similar behavioral responses throughout testing, we believe that the use of i.t. catheters for sustained siRNA delivery did not impact the behavioral assessment of OIH.

### Sustained delivery of MOR-1K exon 13 antisense siRNA decreases MOR-1K gene expression levels

Finally, we evaluated the efficacy of chronic siRNA administration to suppress *MOR-1K* gene expression levels in CXB7/ByJ mice. Given that siRNA was administered intrathecally, we examined *MOR-1K* gene expression within the spinal cord. As predicted, sustained i.t. delivery of antisense siRNA prevented the increase of *MOR-1K* gene expression levels in the spinal cord (Fig 3F; F_(1,11)_ = 29.05, *p* = 0.0002). In contrast, sustained delivery of sense siRNA failed to block increases in *MOR-1K* gene expression levels. These findings suggest that MOR-1K located in spinal sites is necessary for the development of OIH.

## Discussion

Here, we provide the first evidence suggesting that in genetically susceptible animals, MOR-1K contributes to the development of OIH. Compared to C57BL/6J and 129S6 mice, CXB7/ByJ mice exhibited the greatest degree of pain following morphine administration alongside increased *MOR-1K* gene expression levels. Reductions in *MOR-1K* gene expression *via* sustained delivery of antisense siRNA not only prevented OIH, but also unmasked morphine analgesia.

The results of this study demonstrate the significance of genetic variability of the mu-opioid receptor in the development of OIH. Of the three mouse strains, CXB7/ByJ mice exclusively exhibited increased pain sensitivity, evident immediately following morphine administration. Their hyperalgesic pain profile also paralleled increased *MOR-1K* mRNA transcript levels. For this reason, we selected this strain to examine the effects of *MOR-1K* knockdown using antisense exon 13 siRNA. CXB7/ByJ mice receiving sustained i.t. delivery of antisense *MOR-1K* siRNA failed to develop OIH and instead exhibited morphine analgesia. Sustained delivery of antisense siRNA significantly reduced *MOR-1K* gene expression levels within the spinal cord. Given that i.t. delivery provides direct access to the spinal cord, we hypothesize that MOR-1K siRNA integrated into primary and secondary order neurons and cleaved *MOR-1K* mRNA transcripts, resulting in decreased *MOR-1K* gene expression levels.

When examining *MOR-1K* gene expression levels in the three mouse strains, we found that C57BL/6J mice exhibited different gene expression patterns during chronic morphine administration when compared to CXB7/ByJ mice. On day 1, C57BL/6J *MOR-1K* gene expression levels were similar to those of the CXB-7/ByJ mice, but on day 4 their expression levels decreased 2.4% while those of the CXB-7/ByJ mice increased 2.5%. It is possible that *MOR-1K* gene expression is negatively regulated in C57BL/6J mice and that is why they exhibit opioid-induced hyperalgesia to a lesser degree than CXB7/ByJ mice. For example, Lu et al provide evidence of microRNA-103/107 downregulation of mu opioid splice variant MOR-1A [22]. Further research is needed to investigate the possible strain-specific modulators of MOR-1K expression.

In this study, we observed that, despite a return of *MOR-1K* gene expression to baseline levels, C57BL/6J and CXB7/ByJ mice continued to exhibit OIH following morphine cessation (Fig 2). This persistence of OIH following opioid cessation has also been observed in other preclinical and clinical studies. In mice, administration of antagonists against the 5-HT3 receptor (5HT3R) [23], N-Methyl-D-aspartate receptor (NMDAR), melanocortin-1 receptor (MC1R) [24] alongside morphine or beta-2-adrenergic receptor (β2AR) [14] prevent the development of OIH. In addition, β2AR-deficient mice fail to develop OIH [14]. Clinical studies have also shown that administration the β2AR antagonist propranolol [25] or NMDAR antagonist ketamine [26,27] alongside the fast-acting opioid remifentanil hinders the development of OIH following opioid cessation. Collectively, these findings suggest that MOR-1K may interact with other receptors, including 5HT3R, NMDAR, MC1R, and β2AR, to initiate and maintain OIH.

The study highlights the impact of MOR-1 splice variants in opioid and pain signaling. To date, thirty-four MOR-1 splice variants have been identified in mouse, and twenty in human [5], indicating a significant potential for diversity in MOR-1 signaling. MOR-1 splice variants other than MOR-1K have been shown to play modulatory roles in opioid signaling. For example, Liu et al have demonstrated that splice variant MOR-1D heterodimerizes with the gastrin-releasing peptide receptor, resulting in signaling that promotes opioid-induced itch [28]. Another MOR-1 splice variant, MOR-1G, has been shown to heterodimerize with the nociceptin receptor ORL-1 to provide a binding site for the novel opioid analgesic 3-iodobenzoyl-6β-naltrexamide [29]. Along with modulating opioid analgesia and opioid-related side effects, MOR-1 splice variants have also been shown to stabilize the canonical receptor at the cell membrane [30]. These findings demonstrate the influence of splice variants on pain modulation and canonical receptor function. Future research is needed in order to elucidate the functional characteristics of other unexplored MOR-1 splice variants.

Our findings extend previous work examining the effects of genetic variability on opioid analgesia and hyperalgesia. Results from other animal studies have demonstrated strain-specific opioid analgesia efficacy [31] and fentanyl-induced hyperalgesia [32]. Likewise, results from human studies have demonstrated that functional variation in the MOR-1 gene locus regulates opioid responses. For example, the MOR-1 A118G gene polymorphism, which leads to reduced MOR signaling [33], is associated with reduced morphine efficacy [34]. More recently, the *MOR-1K* rs563649 polymorphism, which results in increased *MOR-1K* translation efficiency, has been associated with increased pain sensitivity and blunted morphine efficacy [6]. Combined, these results suggest that genetic susceptibility, particularly in the MOR-1 gene locus, contributes to diminished opioid efficacy and the development of OIH.

In order to understand the mechanisms whereby MOR-1K contributes to OIH, its expression patterns and signaling profiles must be defined. At the tissue level, MOR-1K is expressed in astrocytes within the central nervous system and in perineurial cells within the peripheral nervous system [35], indicating possible influence of glial activation in OIH [4]. At the cellular level, MOR-1K is normally expressed intracellularly [7], where it may drive G_s_-dependent increases in intracellular cAMP and calcium following active transport or passive diffusion of morphine across the cell membrane [36,37]. In the presence of β2AR, a GPCR also implicated in OIH [25]. MOR-1K has been shown to relocate to the cellular membrane *in vitro* [38]. Specifically, the authors utilized immunofluorescence to show that MOR-1K and β2AR co-localize within the cell and that both receptors move to the cell membrane. These data suggest the two receptors interact with one another, perhaps by forming a heterodimer. More *in vitro* studies, such as co-immunoprecipitation, are necessary to determine if this is indeed the case. Although its gene expression and signaling profiles are somewhat established, knowledge on MOR-1K protein expression is lacking and needs to be defined. Currently available molecular tools, however, are not selective enough to identify and isolate the MOR-1K protein. Future MOR-1K studies will benefit from the development of antibodies and ligands that specifically target this splice variant.

In conclusion, the present study demonstrates a functional role for MOR-1K in a murine model of OIH. More work is required to determine MOR-1K’s downstream effectors and signaling mechanisms as well as to understand its contribution to individual variability in a more comprehensive set of opioid responses. Outcomes from present and future studies will help elucidate the neurobiological mechanisms that drive OIH and, in turn, will inform the development of more rational treatment strategies that alleviate clinical pain while reducing OIH risk.

## Supporting Information

S1 ARRIVE Checklist ARRIVE checklist. (DOCX)

S1 Fig Sex-dependent responses to mechanical and thermal heat stimuli across the three strains. Overall, males and females displayed similar behavioral responses to mechanical and thermal heat stimuli within strains. Female C57BL/6J mice demonstrated (A) increased paw withdrawal threshold (F_(9,140)_ = 12.20, *p*<0.0001) and (B) increased responses following repeated exposure to an innocuous mechanical stimulus (F_(9,140)_ = 14.50, *p*<0.0001). Panels A-D: *N =* 7-8/group. Males are represented in black bars while females are represented by white bars. Data expressed as mean ± SEM.****p<0.0001, ***p<0.001, *p<0.05 different from males.
(TIF)

S2 Fig Raw data illustrating behavioral responses of 129S6, C57BL/6J, and CXB7/ByJ mice to mechanical and thermal heat stimuli during chronic morphine administration. All three strains exhibit distinct behavioral differences in (A) paw withdrawal threshold (F_(22,516)_ = 71.94, *p*<0.0001, and when assessing responses to the repeated exposure of (B) an innocuous mechanical stimulus (F_(22,516)_ = 35.37, *p*<0.0001), (C) a noxious mechanical stimulus (F_(22,516)_ = 28.54, *p*<0.0001), and (D) a thermal heat stimulus; F_(22,516)_ = 4.214, *p*<0.0001). Panels A-D: *N* = 15-16/group. Data expressed as mean ± SEM. * *=* different from baseline.
(TIF)

S3 Fig Raw data illustrating behavioral responses of 129S6, C57BL/6J, and CXB7/ByJ mice to mechanical and thermal heat stimuli during saline administration. Strains exhibit no significant changes from their respective baselines when assessing for (A) paw withdrawal threshold (F_(2,516)_ = 377.7, *p*<0.0001), and when assessing responses following repeated exposure to (B) an innocuous (F_(2,516)_ = 29.08, *p*<0.0001), or (C) noxious mechanical stimulus (F_(2,516)_ = 1857, *p*<0.0001). (D) Unlike 129S6 and C57BL/6J mice, CXB7/ByJ mice exhibited increased responses to thermal heat stimuli (F_(2,516)_ = 115.2, *p*<0.0001) starting on day 1 following saline administration that steadily returned to baseline throughout testing. Panels A-D: *N =* 15-16/group. Data expressed as mean ± SEM. * = different from baseline.
(TIF)

S4 Fig Z-scores of *MOR-1K* gene expression levels in discrete tissues of 129S6 mice. Tissue samples from (A) spinal cord, (B) medulla, (C) pons, (D) periaqueductal gray, (E) thalamus, (F) hypothalamus, (G) striatum, (H) nucleus accumbens, and (I) frontal lobe have similar *MOR-1K* gene expression levels in 129S6 mice. Panels A-I: *N =* 7/group. Data expressed as Z-score.
(TIF)

S5 Fig Z-scores of *MOR-1K* gene expression levels in discrete brain regions of C57BL/6J mice. Tissue samples from (A) spinal cord, (B) medulla, (C) pons, (D) periaqueductal gray, (E) thalamus, (F) hypothalamus, (G) striatum, (H) nucleus accumbens, and (I) frontal lobe have similar *MOR-1K* gene expression levels in C57BL/6J mice. Panels A-I: *N =* 7/group. Data expressed as Z-score.
(TIF)

S6 Fig Z-scores of *MOR-1K* gene expression levels in discrete brain regions of CXB7/ByJ mice. Tissue samples from (A) spinal cord, (B) medulla, (C) pons, (D) periaqueductal gray, (E) thalamus, (F) hypothalamus, (G) striatum, (H) nucleus accumbens, and (I) frontal lobe have similar *MOR-1K* gene expression levels in CXB7/ByJ mice. Panels A-I: *N =* 7/group. Data expressed as Z-score.
(TIF)

S7 Fig Relative quantification of other exon 11 MOR-1 splice variants (A-C). Chronic morphine administration does not significantly alter the gene expression levels of MOR-1 splice variants that contain exon 11, exon 14, or exon 16. Panels A-I: *N =* 7-8/group. Data expressed as Z-score.
(TIF)

S8 Fig Sex-dependent behavioral responses to mechanical stimuli across Antisense, Sense, and Sham mice. Male and female Antisense mice (A-C), Sense mice (D-F), and Sham mice (G-I) exhibited similar responses to mechanical stimuli within their respective treatment groups. Panels A-I: *N* = 3-4/group. Data expressed as mean ± SEM.
(TIF)

S9 Fig Raw data illustrate behavioral responses of Antisense, Sense, and Sham mice to mechanical stimuli during chronic morphine administration. Antisense mice exhibit (A) lower paw withdrawal threshold, (B) decreased responses to repeated innocuous (C) and noxious stimuli when compared to Sense and Sham mice. Panels A-D: *N =* 6-9/group. Data expressed as mean ± SEM. * = different from baseline.
(TIF)

S10 Fig Raw data illustrate behavioral responses of Antisense, Sense, and Sham mice to mechanical stimuli during saline administration. Antisense, Sense, and Sham mice did not exhibit behavioral differences from their respective baselines when assessing for (A) paw withdrawal threshold (F_(2,160)_ = 34.87, *p*<0.0001), (B) mechanical allodynia (F_(2,160)_ = 45.01, *p*<0.0001), (C) mechanical hyperalgesia (F_(2,160)_ = 23.43, *p*<0.0001) during saline administration. Panels A-D: *N =* 6-9/group. Data expressed as mean ± SEM. * = different from baseline.
(TIF)
