# Supplementary material for: Mu Opioid Splice Variant MOR-1K Contributes to the Development of Opioid-Induced Hyperalgesia
Source: PLoS One. 2015 Aug 13;10(8):e0135711. doi: 10.1371/journal.pone.0135711 (PMC4535978; doi:10.1371/journal.pone.0135711)

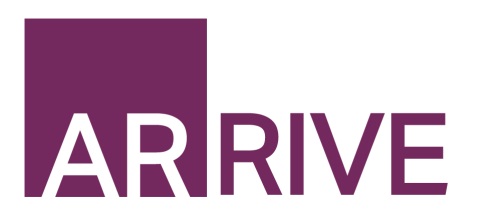


The ARRIVE Guidelines Checklist

Animal Research: Reporting In Vivo Experiments

Carol Kilkenny^1^, William J Browne^2^, Innes C Cuthill^3^, Michael Emerson^4^ and Douglas G Altman^5^

*^1^The National Centre for the Replacement, Refinement and Reduction of Animals in Research, London, UK, ^2^School of Veterinary Science, University of Bristol, Bristol, UK, ^3^School of Biological Sciences, University of Bristol, Bristol, UK, ^4^National Heart and Lung Institute, Imperial College London, UK, ^5^Centre for Statistics in Medicine, University of Oxford, Oxford, UK.*

|  | | ITEM | RECOMMENDATION | Section/ Paragraph |
| --- | --- | --- | --- | --- |
| 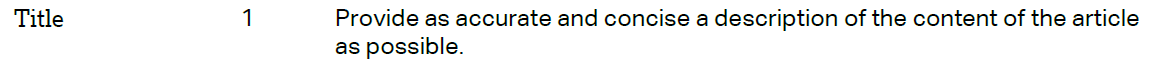 | | | Title |  |
| 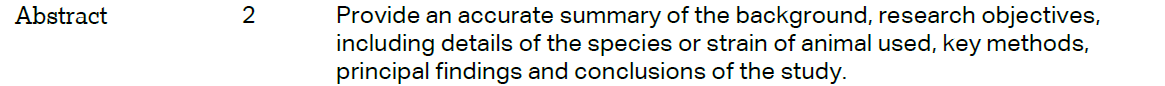 | | | Abstract |  |
| INTRODUCTION | | |  |  |
| 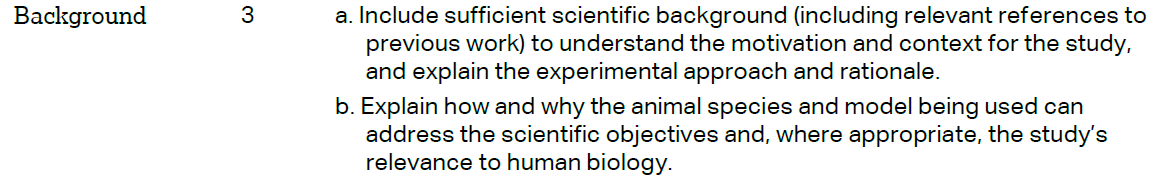 | | | Paragraph 1-2 |  |
| 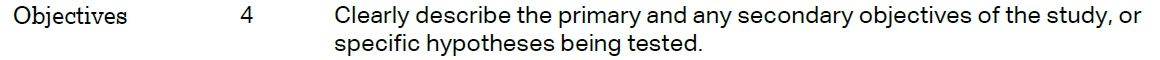 | | | Paragraph 3 |  |
| METHODS | | |  |  |
| 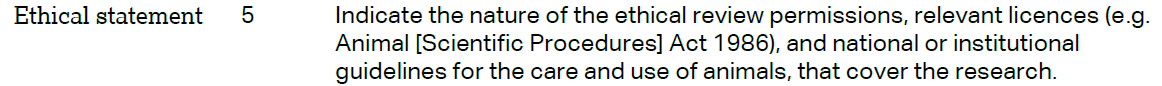 | | | Paragraph 1 |  |
| 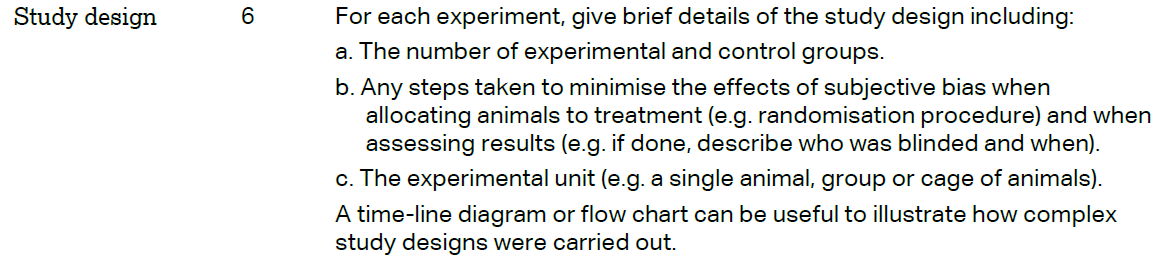 | | | A-C: Paragraphs4, 5  Timelines:  Fig 2A, 3A |  |
| 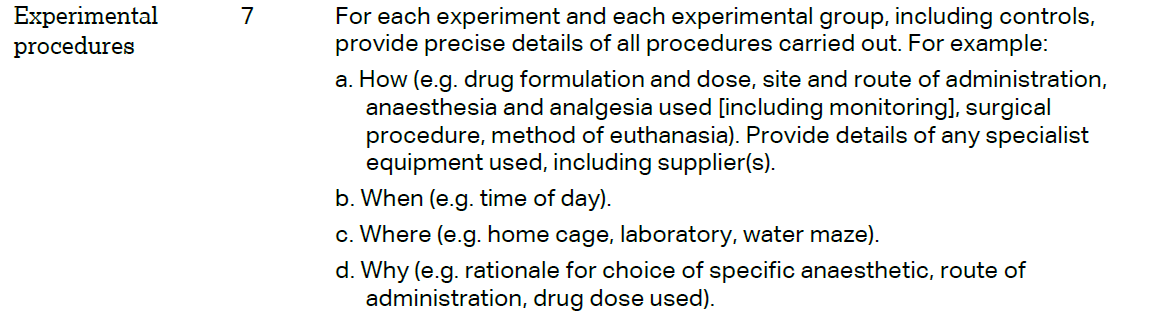 | | | A-B: Paragraph 3-8  Surgical Procedure:  Paragraph 5 |  |
| 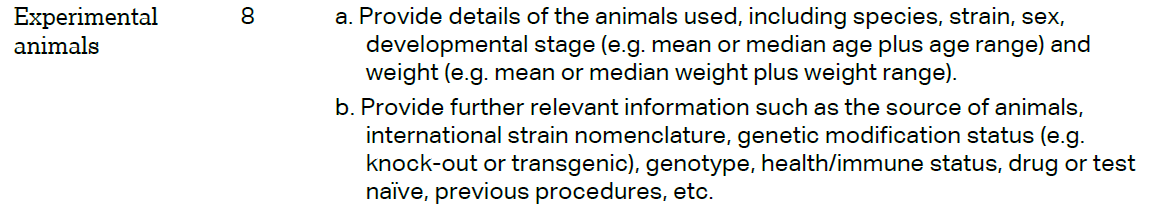 | | | Paragraph 2 |  |

| 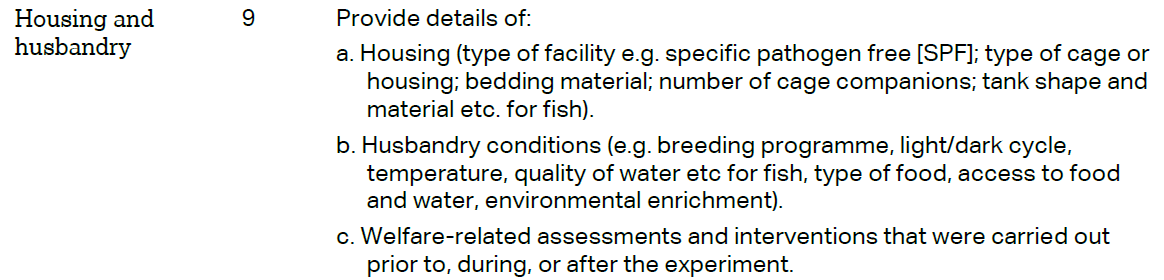 | Paragraph 2 | |
| --- | --- | --- |
| 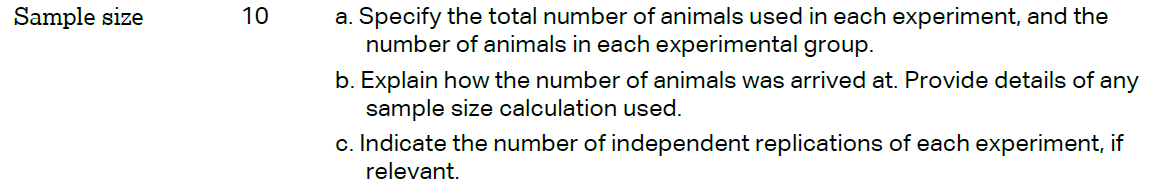 | Paragraphs 4,5 | |
| 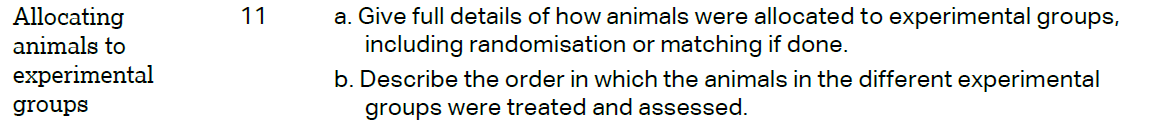 | Paragraph 4,5 | |
| 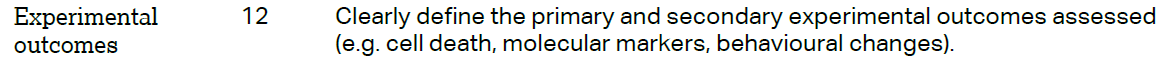 | Paragraph 4,5 | |
| 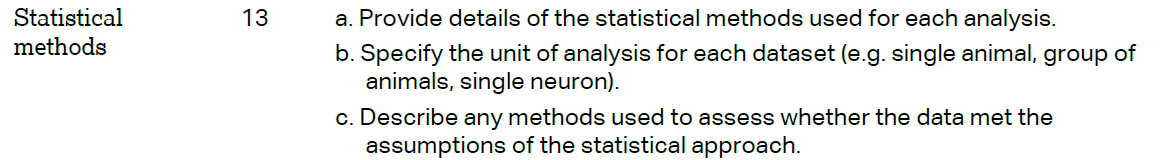 | Paragraph 9 | |
| RESULTS |  | |
| 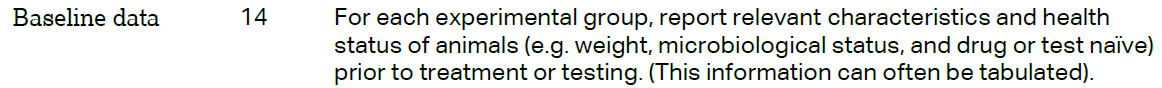 | Paragraph 1  Figure 1; S3 Fig.; S10 Fig. | |
| 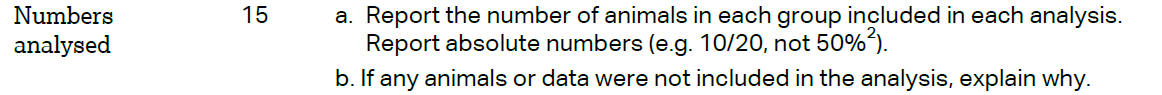 | Methods  Paragraphs 3, 9,12 | |
| 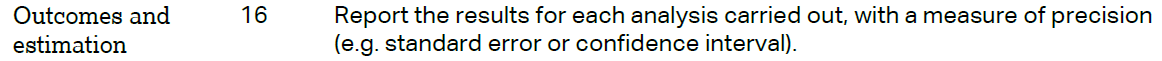 | Paragraphs 2-12  Figures 2,3 | |
| 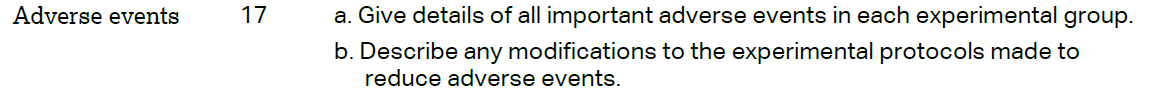 |  | |
| DISCUSSION |  | |
| 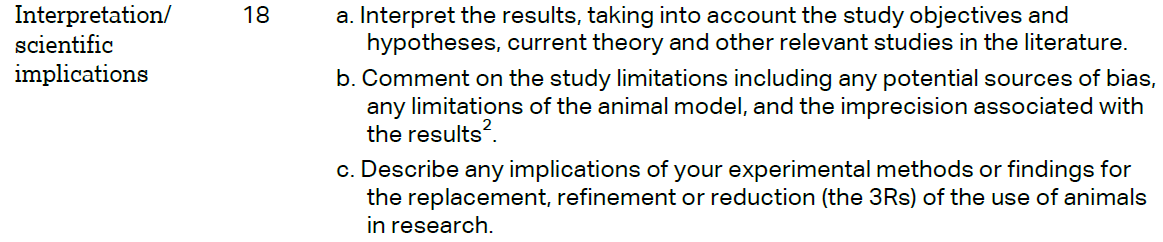 | Throughout  Paragraphs 1-4 | |
| 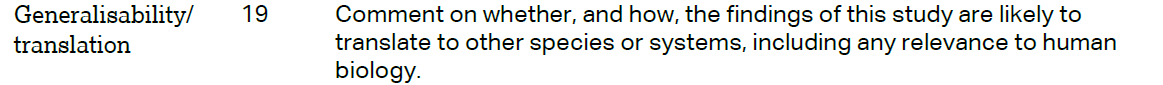 | Paragraphs 4-6 | |
| 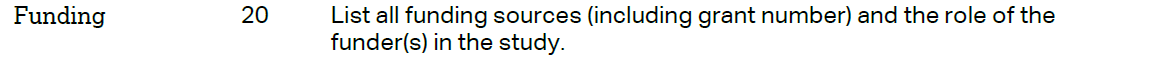 | |  |


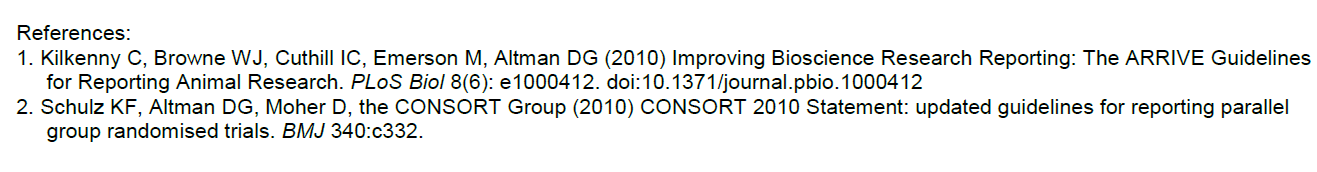

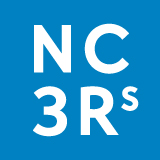

Supplement: S1 ARRIVE Checklist — (DOCX) [file pone.0135711.s001.docx]
