# Supplementary figures and images for: Mu Opioid Splice Variant MOR-1K Contributes to the Development of Opioid-Induced Hyperalgesia
Source: PLoS One. 2015 Aug 13;10(8):e0135711. doi: 10.1371/journal.pone.0135711 (PMC4535978; doi:10.1371/journal.pone.0135711)

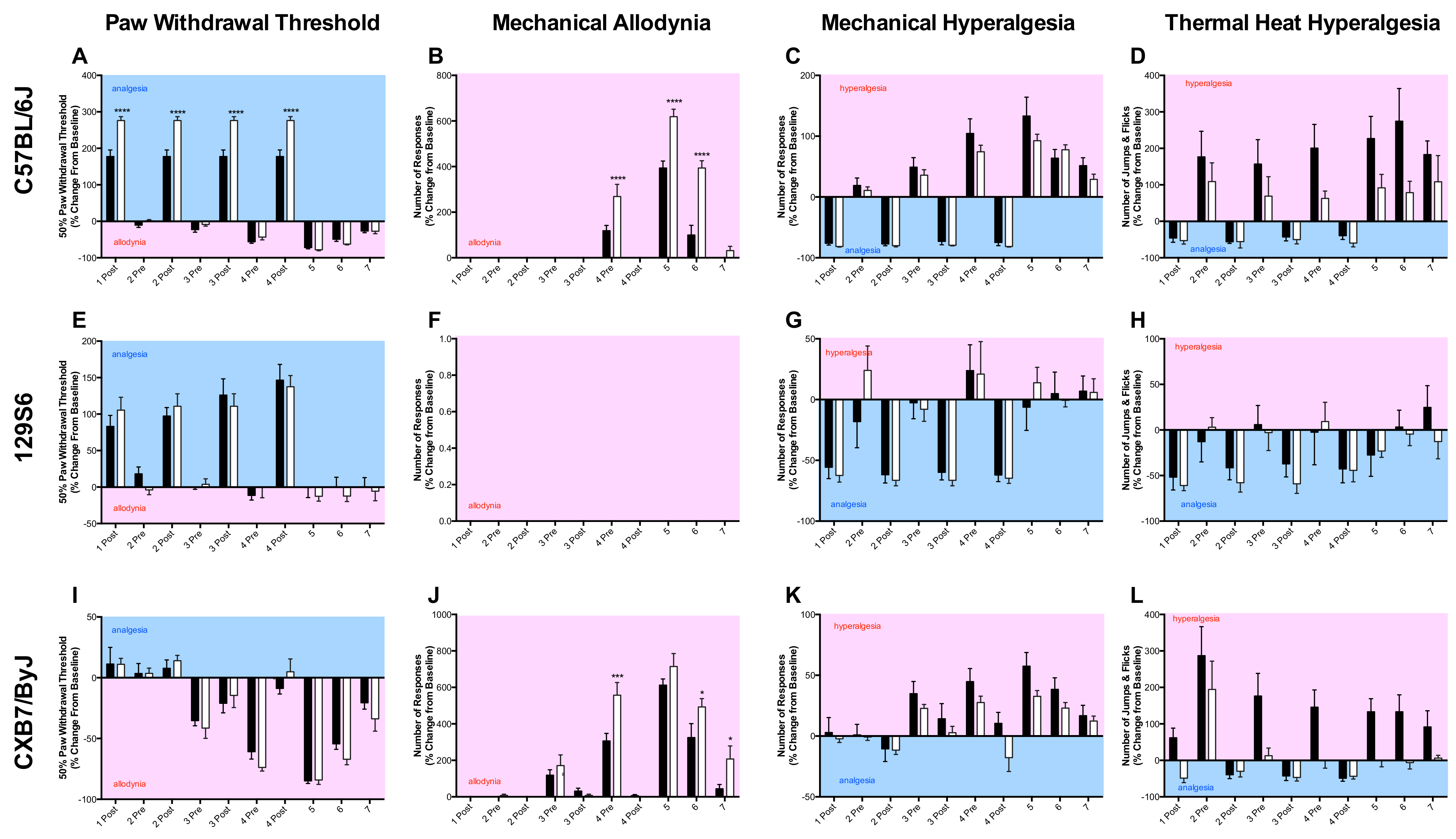

Supplement: S1 Fig — Overall, males and females displayed similar behavioral responses to mechanical and thermal heat stimuli within strains. Female C57BL/6J mice demonstrated (A) increased paw withdrawal threshold (F(9,140) = 12.20, p<0.0001) and (B) increased responses following repeated exposure to an innocuous mechanical stimulus (F(9,140) = 14.50, p<0.0001). Panels A-D: N = 7-8/group. Males are represented in black bars while females are represented by white bars. Data expressed as mean ± SEM.****p<0.0001, ***p<0.001, *p<0.05 different from males. (TIF) [file pone.0135711.s002.tif]

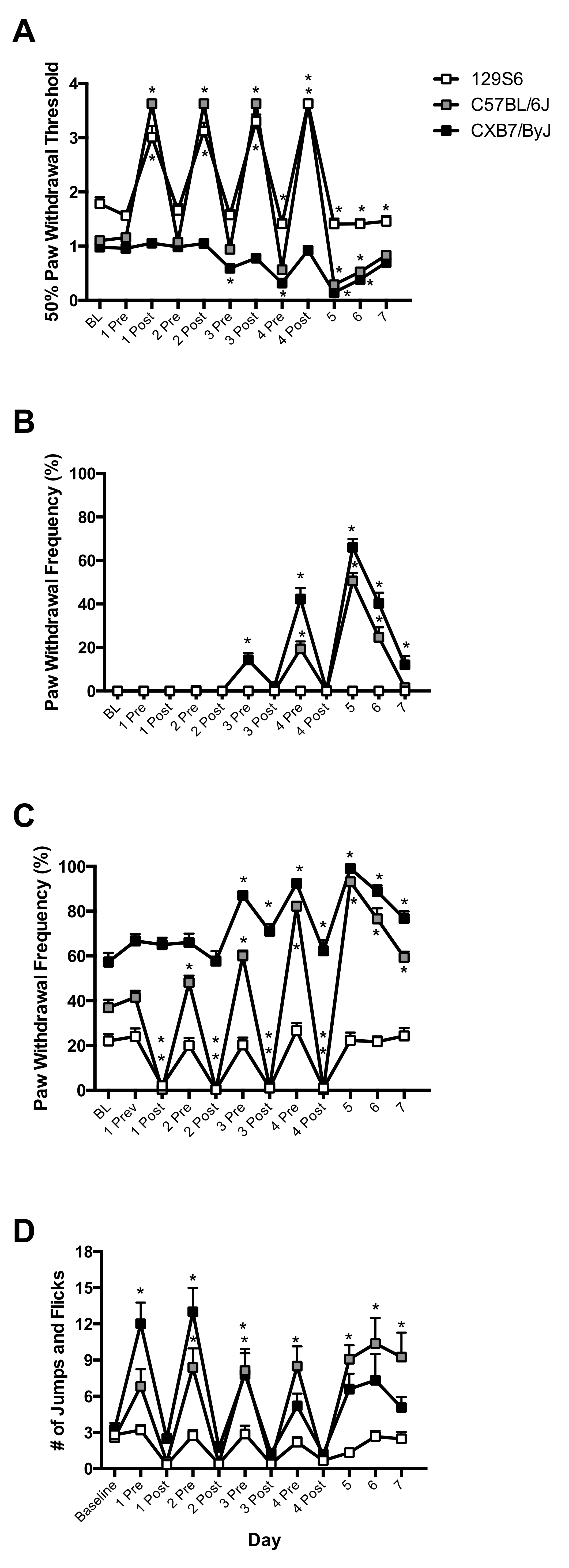

Supplement: S2 Fig — All three strains exhibit distinct behavioral differences in (A) paw withdrawal threshold (F(22,516) = 71.94, p<0.0001, and when assessing responses to the repeated exposure of (B) an innocuous mechanical stimulus (F(22,516) = 35.37, p<0.0001), (C) a noxious mechanical stimulus (F(22,516) = 28.54, p<0.0001), and (D) a thermal heat stimulus; F(22,516) = 4.214, p<0.0001). Panels A-D: N = 15-16/group. Data expressed as mean ± SEM. * = different from baseline. (TIF) [file pone.0135711.s003.tif]

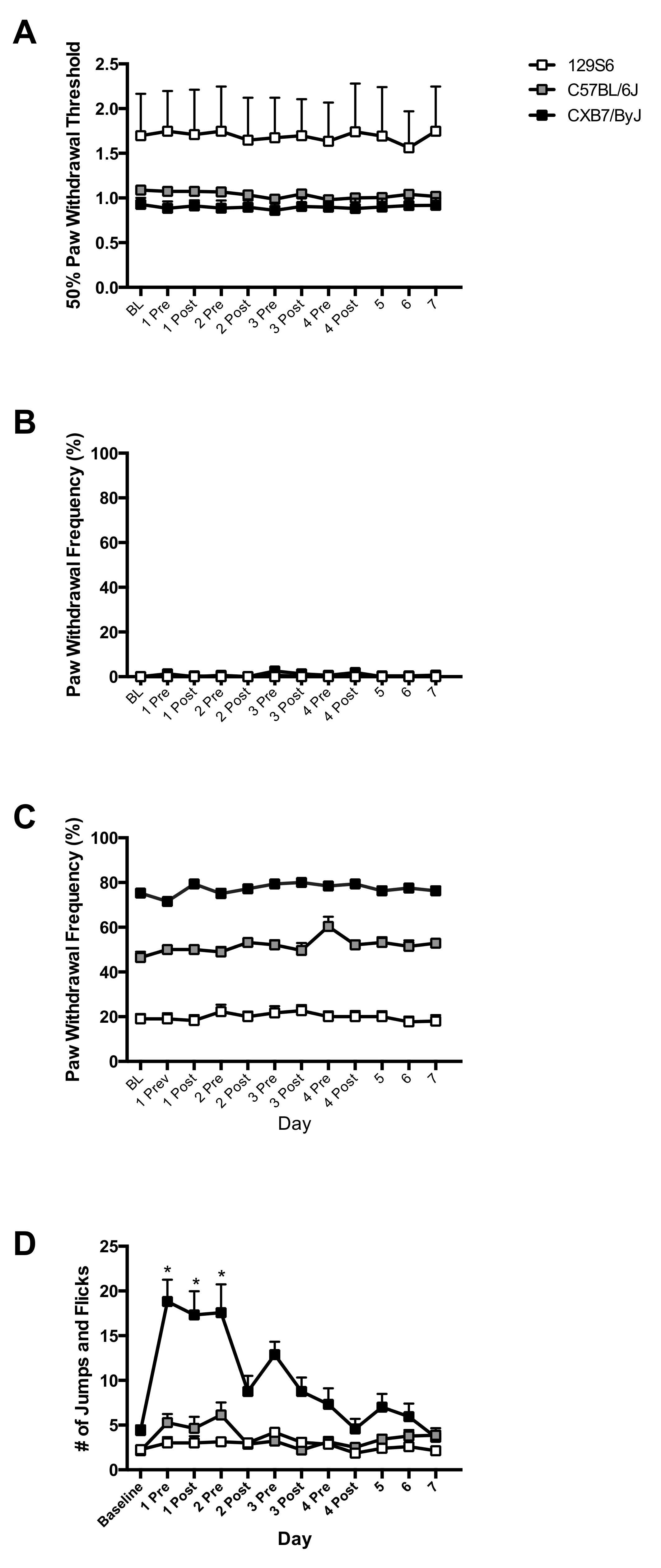

Supplement: S3 Fig — Strains exhibit no significant changes from their respective baselines when assessing for (A) paw withdrawal threshold (F(2,516) = 377.7, p<0.0001), and when assessing responses following repeated exposure to (B) an innocuous (F(2,516) = 29.08, p<0.0001), or (C) noxious mechanical stimulus (F(2,516) = 1857, p<0.0001). (D) Unlike 129S6 and C57BL/6J mice, CXB7/ByJ mice exhibited increased responses to thermal heat stimuli (F(2,516) = 115.2, p<0.0001) starting on day 1 following saline administration that steadily returned to baseline throughout testing. Panels A-D: N = 15-16/group. Data expressed as mean ± SEM. * = different from baseline. (TIF) [file pone.0135711.s004.tif]

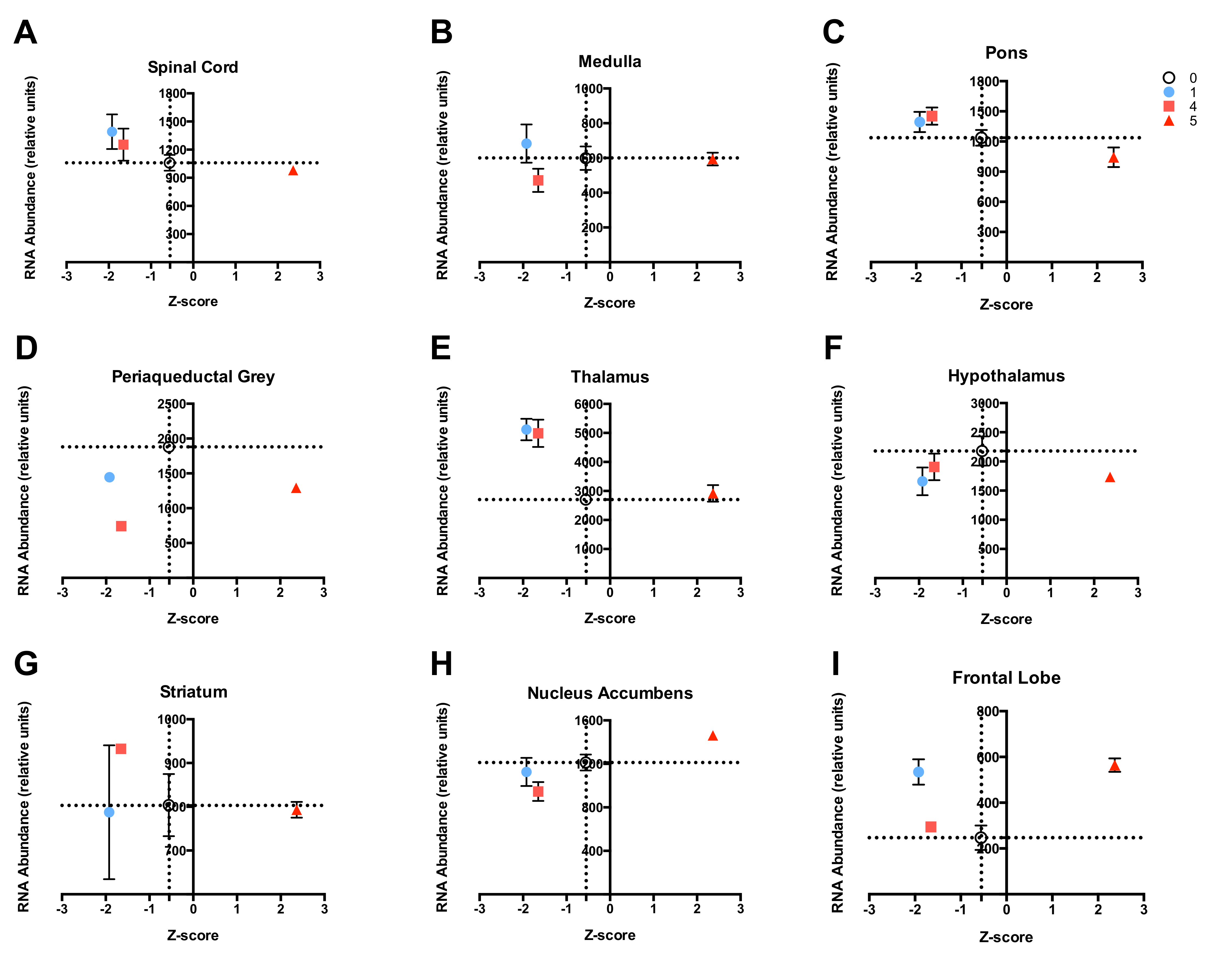

Supplement: S4 Fig — Tissue samples from (A) spinal cord, (B) medulla, (C) pons, (D) periaqueductal gray, (E) thalamus, (F) hypothalamus, (G) striatum, (H) nucleus accumbens, and (I) frontal lobe have similar MOR-1K gene expression levels in 129S6 mice. Panels A-I: N = 7/group. Data expressed as Z-score. (TIF) [file pone.0135711.s005.tif]

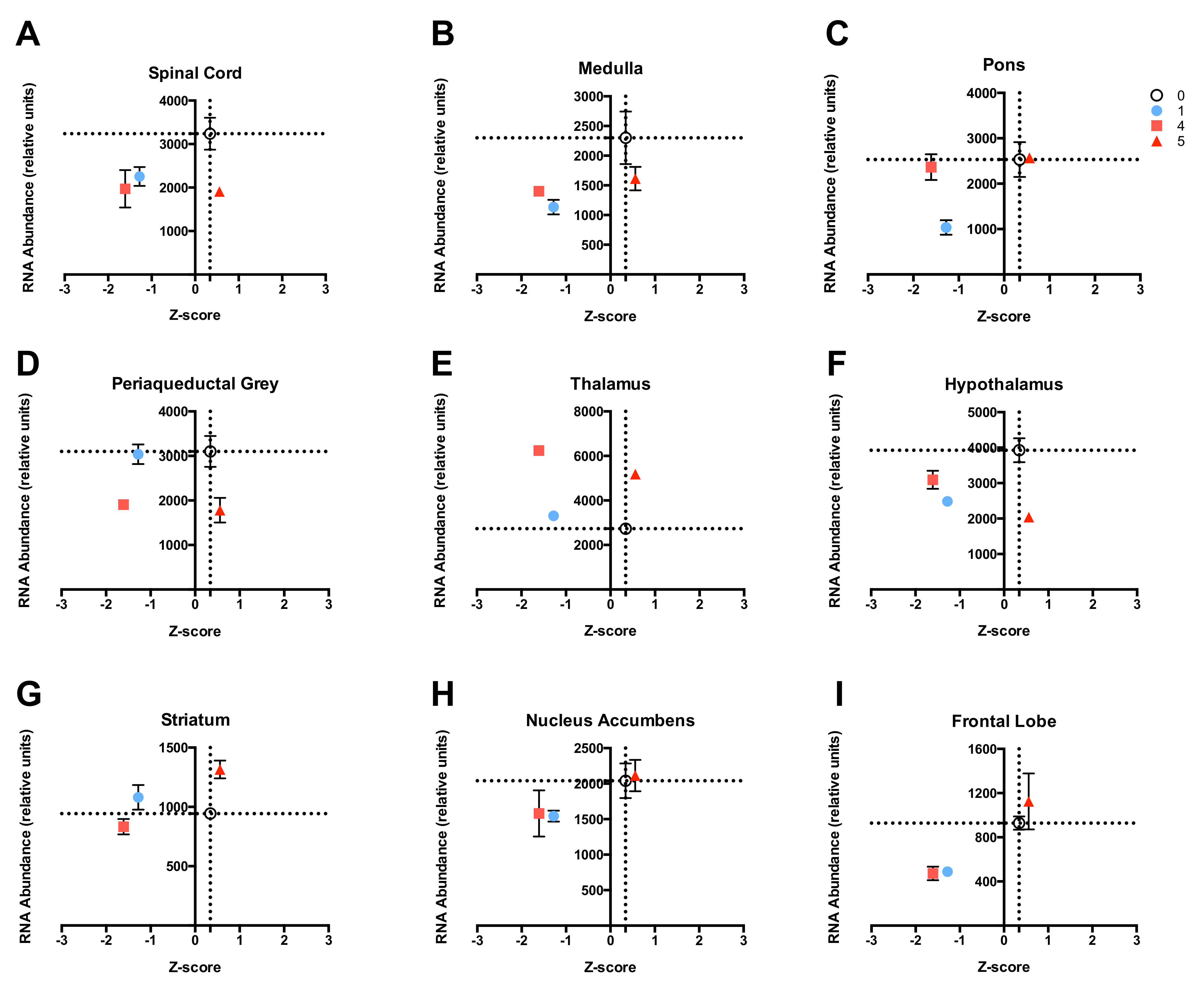

Supplement: S5 Fig — Tissue samples from (A) spinal cord, (B) medulla, (C) pons, (D) periaqueductal gray, (E) thalamus, (F) hypothalamus, (G) striatum, (H) nucleus accumbens, and (I) frontal lobe have similar MOR-1K gene expression levels in C57BL/6J mice. Panels A-I: N = 7/group. Data expressed as Z-score. (TIF) [file pone.0135711.s006.tif]

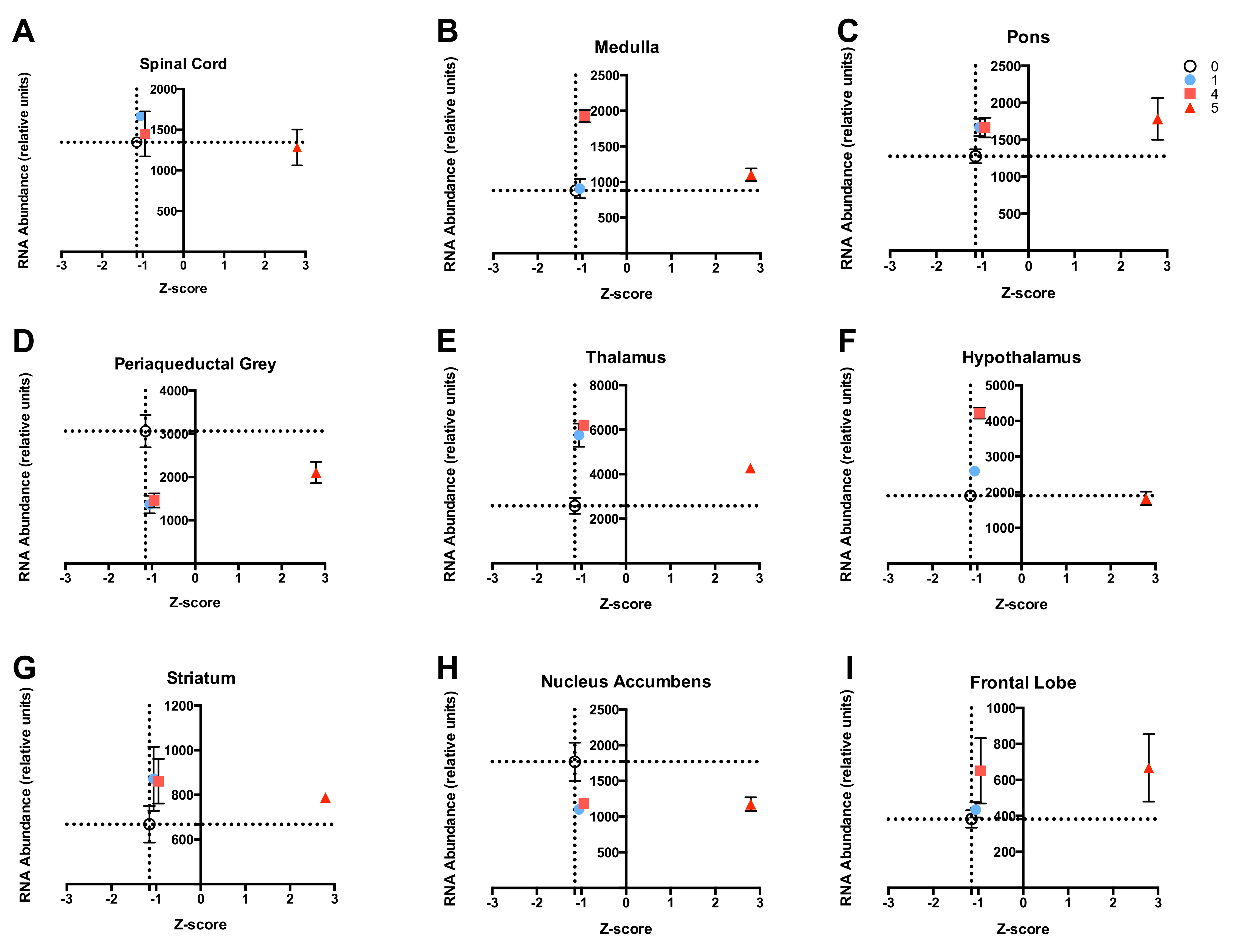

Supplement: S6 Fig — Tissue samples from (A) spinal cord, (B) medulla, (C) pons, (D) periaqueductal gray, (E) thalamus, (F) hypothalamus, (G) striatum, (H) nucleus accumbens, and (I) frontal lobe have similar MOR-1K gene expression levels in CXB7/ByJ mice. Panels A-I: N = 7/group. Data expressed as Z-score. (TIF) [file pone.0135711.s007.tif]

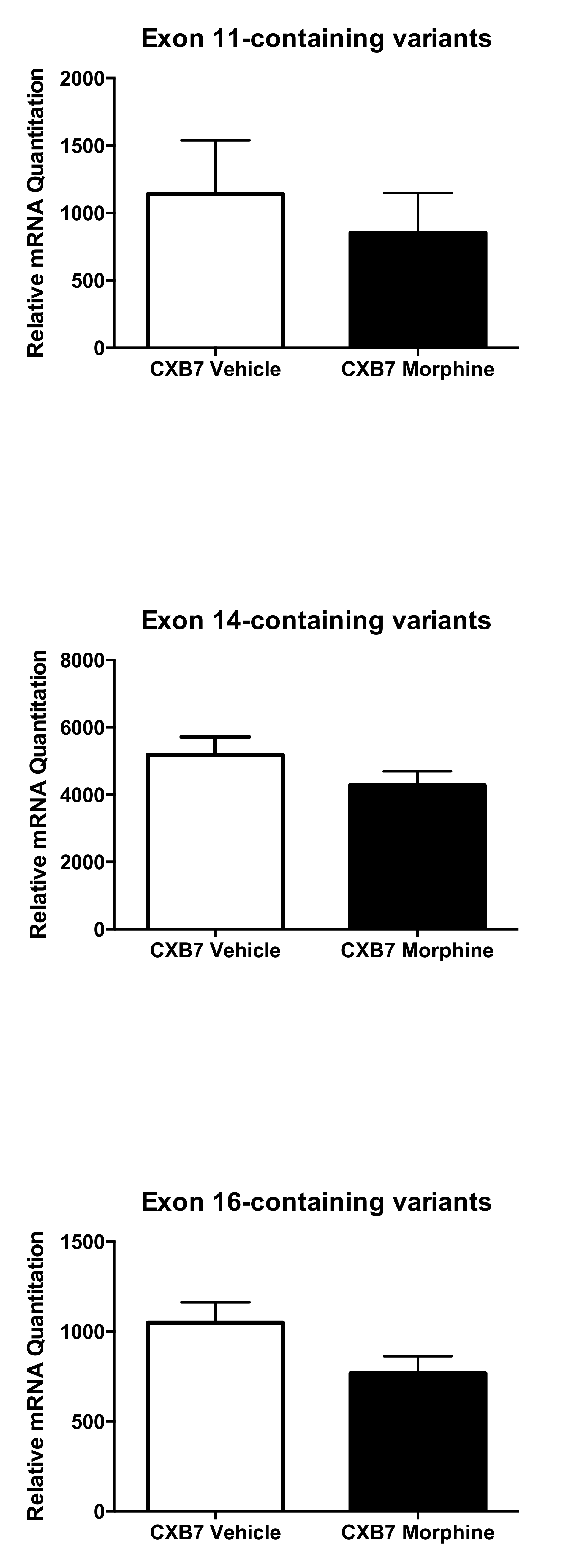

Supplement: S7 Fig — Chronic morphine administration does not significantly alter the gene expression levels of MOR-1 splice variants that contain exon 11, exon 14, or exon 16. Panels A-I: N = 7-8/group. Data expressed as Z-score. (TIF) [file pone.0135711.s008.tif]

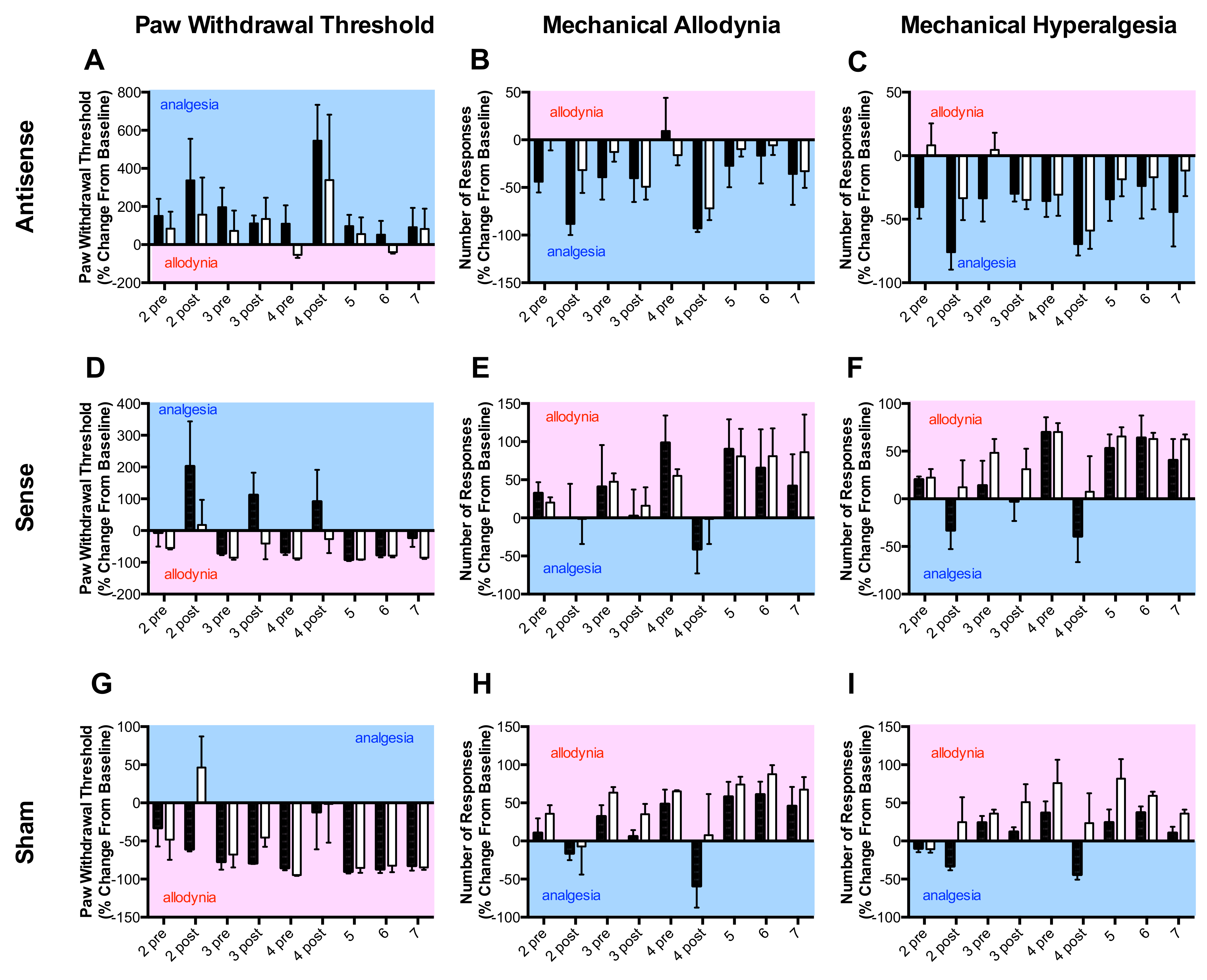

Supplement: S8 Fig — Male and female Antisense mice (A-C), Sense mice (D-F), and Sham mice (G-I) exhibited similar responses to mechanical stimuli within their respective treatment groups. Panels A-I: N = 3-4/group. Data expressed as mean ± SEM. (TIF) [file pone.0135711.s009.tif]

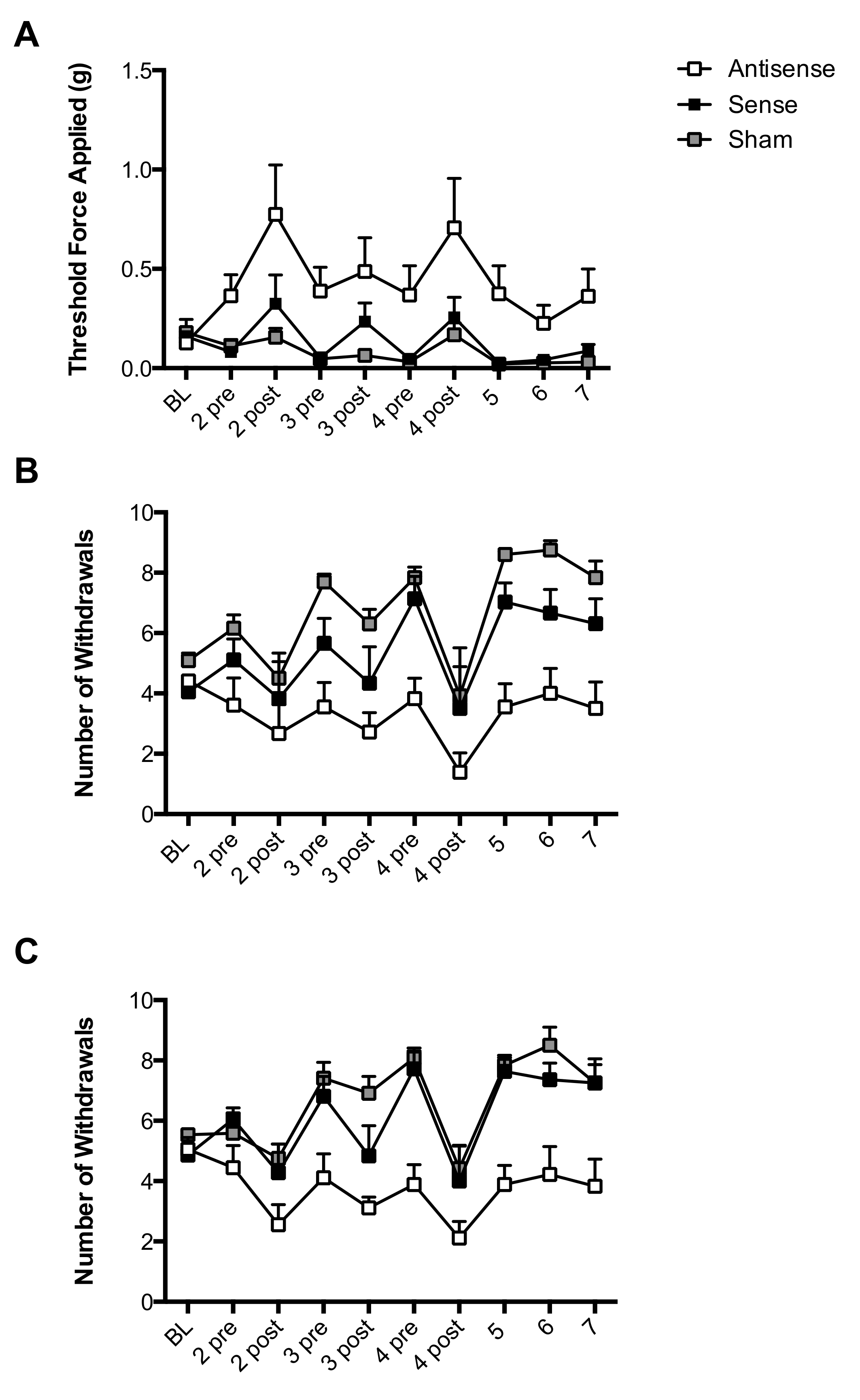

Supplement: S9 Fig — Antisense mice exhibit (A) lower paw withdrawal threshold, (B) decreased responses to repeated innocuous (C) and noxious stimuli when compared to Sense and Sham mice. Panels A-D: N = 6-9/group. Data expressed as mean ± SEM. * = different from baseline. (TIF) [file pone.0135711.s010.tif]

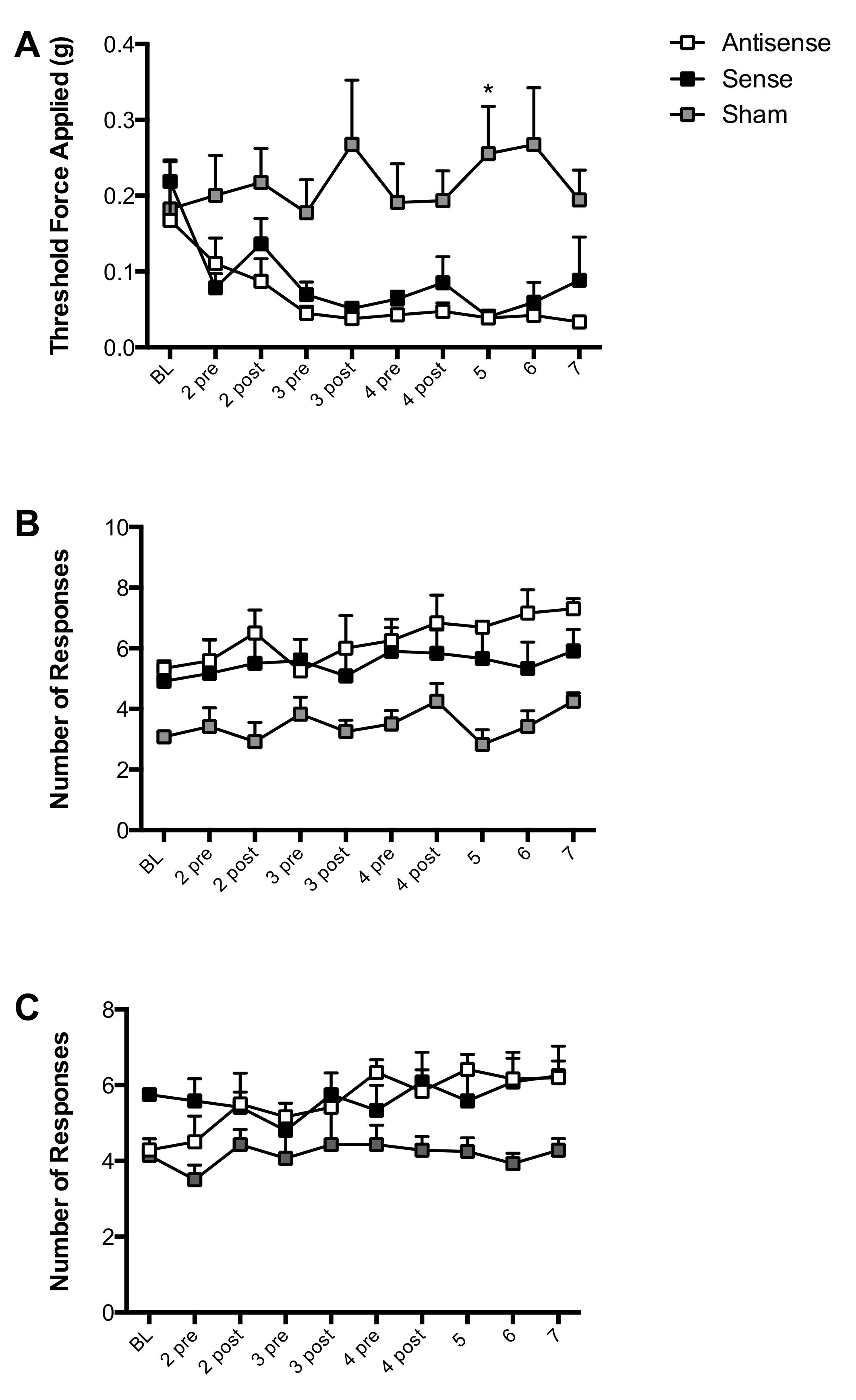

Supplement: S10 Fig — Antisense, Sense, and Sham mice did not exhibit behavioral differences from their respective baselines when assessing for (A) paw withdrawal threshold (F(2,160) = 34.87, p<0.0001), (B) mechanical allodynia (F(2,160) = 45.01, p<0.0001), (C) mechanical hyperalgesia (F(2,160) = 23.43, p<0.0001) during saline administration. Panels A-D: N = 6-9/group. Data expressed as mean ± SEM. * = different from baseline. (TIF) [file pone.0135711.s011.tif]
